# Optimizing the management of venous disease: Expert consensus from a multinational panel of phlebology specialists

**DOI:** 10.1016/j.jvsv.2026.102593

**Published:** 2026-08-04

**Authors:** Sergiu-Ciprian Matei, Nadelin Nikolov, Miroslava Popović, Vasyl Shaprynskyi, Surat Temirov, Vassil Chervenkoff, Yoann Molinard, Julie Escola, Nenad Ilijevski

**Affiliations:** a Abdominal Surgery and Phlebology Research Center, Victor Babeş University of Medicine and Pharmacy Timişoara, Timişoara, Romania; b 1’st Surgical Department, “Pius Brînzeu” County Emergency Clinical Hospital Timişoara, Timişoara, Romania; c Vascular Surgery Department, National Heart Hospital, Sofia, Bulgaria; d Vascular Surgery Department, CHC Zvezdara, Belgrade, Serbia; e Department of Mini-invasive Surgery, State Institution of Science “Center of Innovative Technologies” State Administrative Department, Kyiv, Ukraine; f Vascular Surgery Department, Central Hospital Ministry of Internal Affairs Author Republic of Uzbekistan, Tashkent, Uzbekistan; g Vascular Surgery Department, Acibadem Citiy Clinic UMHAT Tokuda, Sofia, Bulgaria; h Laboratoire Innotech International, Groupe Innothera, Arcueil, France; i Vascular Surgery Department, School of Medicine Belgrade University, Dedinje Cardiovascular Institute, Belgrade, Serbia

**Keywords:** Chronic venous disease, Varicose veins, Expert consensus, Venoactive drugs, Compression therapy, Endovenous ablation

## Abstract

Chronic venous disease and varicose veins (VV) are highly prevalent yet remain inconsistently managed despite existing guidelines, with variability in treatment selection, patient stratification, and use of conservative therapies. This multinational initiative sought to establish expert consensus on key clinical questions in venous disease management. A Delphi-like process involved 38 phlebologists from 15 countries across Europe, North Africa, and Central Asia. Four thematic workshops addressed interventional and conservative approaches for both chronic venous disease and VV. Twenty predefined questions were discussed, and resulting statements were rated on a 5-point Likert scale, with consensus defined as ≥75% agreement. Of 34 proposed statements, 30 (88%) achieved consensus. The panel emphasized that treatment decisions should be driven primarily by symptoms and quality of life rather than anatomy alone. Endovenous procedures were supported for symptomatic disease, with endovenous laser ablation and radiofrequency ablation considered equivalent for VV. Diosmin-based venoactive drugs, often combined with compression, were endorsed across Clinical, Etiologic, Anatomic, and Pathophysiologic classes for symptom relief, although adherence to conservative therapy remains limited. This consensus provides practical guidance for individualized care and highlights unmet needs in risk stratification, adherence, and long-term comparative outcomes.

Chronic venous disease (CVD), including varicose veins (VV) and more advanced forms of chronic venous insufficiency, is a common and progressive condition affecting a substantial proportion of the adult population, with prevalence estimates varying according to definitions, diagnostic methods, and populations studied.^1^, ^2^, ^3^, ^4^, ^5^ Its clinical spectrum ranges from visible varicosities to leg heaviness, pain, edema, skin changes, and venous ulcers, all of which may impair quality of life (QoL), work productivity, and daily activities.^1^, ^2^, ^3^^,^^6^^,^^7^ The economic burden is considerable, particularly in patients requiring repeated interventions or treatment for advanced disease and complications.^1^^,^^8^

Several risk factors have been consistently associated with CVD, including increasing age, female sex, family history, genetic factors pregnancy, obesity, sedentary lifestyle, prolonged standing or sitting, previous thrombophlebitis, and leg trauma.^1^^,^^3^^,^^6^^,^^9^, ^10^, ^11^ Although CVD is widely prevalent, its diagnosis and management remain heterogeneous across countries, health care systems, and medical specialties.^3^^,^^4^,^6^,^12^^,^^13^ The Clinical, Etiologic, Anatomic, and Pathophysiologic (CEAP) classification provides a standardized framework to describe the CEAP features of CVD, with clinical classes ranging from C0 to C6; VV are classified as C2, whereas more advanced disease includes edema, skin changes, and active or healed venous ulceration.^14^^,^^15^

Despite available national and international guidelines, several aspects of CVD and VV management remain debated, including the role and timing of compression therapy, the indications for venoactive drugs (VADs), treatment strategies in elderly or high-risk patients, the selection of surgical or endovenous techniques, and the prevention and management of recurrence.^12^^,^^13^^,^^16^^,^^17^ In areas where evidence is limited, heterogeneous, or difficult to apply across different health care settings, structured expert consensus can help support clinical decision-making.

The objective of this study was to develop consensus-based recommendations on selected clinical questions related to the management of CVD and VV. To achieve this, we used a structured, modified Delphi-based consensus process involving a multinational panel of phlebology specialists. This approach was designed to capture expert agreement while allowing predefined adaptations to the classical Delphi methodology, including the selection of clinically relevant statements and iterative evaluation by the expert panel.

## Methods

### Consensus design

A Delphi-like consensus approach was used, combining structured expert discussion with real-time anonymous voting via Slido, an interactive digital platform. The goal was to gather expert opinions on challenging clinical questions regarding the management of CVD and VV, and to formulate consensus-based recommendations to contribute to enhanced clinical practice.

For this purpose, a scientific steering committee composed of five experienced phlebology experts was established to supervise the consensus process and identify knowledge gaps in the management of CVD and VV. The steering committee discussed the need to define four themes in venous disease in emerging economies based on literature data.

### Consensus process overview

The consensus process was conducted in four structured steps during a dedicated scientific meeting that brought together 40 experts in phlebology from 15 countries across regions/areas of three continents (such as Eastern Europe, North Africa, and Central and Southeast Asia). These experts were invited to participate in a structured, in-person consensus-building process based on thematic workshop discussions. One workshop was held per thematic area, allowing for focused, small-group exchanges ahead of a full plenary vote.

The first step consisted of identifying the discussion areas. Four workshop themes were selected by the steering committee to address the following areas of clinical uncertainty in the management of CVD and VV.

1. Surgical and interventional management of CVD
2. Medical and conservative management of CVD
3. Surgical and interventional management of VV
4. Medical and conservative management of VV

To support the development of clinically relevant questions for each thematic workshop, a targeted literature review using the PubMed database was conducted focusing on the four predefined themes as listed above. Keyword combinations and Boolean operators were adapted to each of the four thematic domains.

Studies were selected for their relevance to common clinical dilemmas in the real-world practice and their potential to inform expert discussion. The insights gained from this narrative review were then used to draft clinical questions. Finally, 20 questions were formulated and validated by the scientific committee to serve as the basis for the consensus process (Table I, Table II, Table III, Table IV).

**Table I:** Workshop 1 (W1): Surgical and interventional management of chronic venous disease (CVD)—consensus statements, voting results, and key discussion points

Predefined questions	Statements	Vote % (n/N)		Key discussion points
Question 1 – W1Q1: How do you decide among hybrid, open surgery, and endovascular techniques in patients with post-thrombotic syndrome (PTS)?	**Patients classified as CEAP C4-C6 should be considered for intervention. Patients with C3 classification and severe symptoms may also be eligible.**	**✓**	**94** (30/32)	- Endovascular, open surgical, and hybrid procedures can all be appropriate depending on the anatomical extent and clinical severity of the disease.
	**The localization and length of venous lesions are key criteria in selecting the appropriate interventional technique.**	**✓**	**88** (28/32)	
	**Isolated iliac vein lesions should be managed exclusively through endovascular techniques.**	**✓**	**97** (30/31)	
Question 2 – W1Q2: Are there patient populations for whom intervention should be avoided?	All patients are eligible for endovascular surgery, taking into account specific contraindications (eg, hypersensitivity, high creatinine level) and possible elderly anatomical and pathomorphological damages.	X	71 (22/31)	- Importance of risk stratification, particularly in elderly or comorbid populations were emphasized. - Slight divergence on endovascular eligibility criteria.
	**EuroSCORE should be used as a tool to evaluate the likelihood benefit and point of risk stratification for hybrid or open surgery.**	**✓**	**83** (25/30)	
Question 3 – W1Q3: What is the alternative for those patients excluded from surgical or interventional procedures?	**Conservative treatment with venoactive drugs and compression therapy, along with lifestyle modifications such as off-loading techniques, should be recommended for patients who are not considered candidates for surgical intervention based on EuroSCORE risk stratification**.	**✓**	**93** (28/30)	- Multimodal conservative strategy (such as diosmin-based agents, compression, and reduced standing time) aims to alleviate symptoms and slow disease progression in patients when surgical risks outweigh benefits
Question 4 – W1Q4: Which anatomical or clinical criteria most influence your treatment choice?	**Severity of symptoms and impact on QoL should guide the decision to intervene or not.**	**✓**	**97** (30/31)	- Importance of a patient-oriented approach, prioritizing clinical presentation (eg, symptom burden and functional impairment) over anatomical findings alone. - Imaging remains essential for procedural planning; decision to proceed with intervention should ultimately consider the individual's QoL and symptomatology.
	**Duplex ultrasound and computed tomography (CT) venography should be used to determine the most appropriate procedure.**	**✓**	**97** (30/31)	
Question 5 – W1Q5: How do you define a successful outcome after surgery or intervention in patients with PTS?	**The procedure should be free of complications.**	**✓**	**77** (24/31)	- Key outcome criteria cited: safety, symptom relief, and QoL improvements, as well as the role of CEAP progression in follow-up evaluation. - Slight divergence regarding the optimal timeline for postprocedural assessment, with suggestions for more flexibility based on national practices or patient-specific factors.
	**The main aim of the intervention is to improve QoL; subjective symptoms and QoL evolution should be evaluated using objective scoring systems.**	**✓**	**100** (30/30)	
	CEAP classification changes serve as a basis for evaluating postprocedural outcomes and patient condition.	X	74 (23/31)	
	**Successful outcomes should be assessed at short term (<1 month), mid-term (3-6 months), and long term (>6 months).**	**✓**	**97** (30/31)	

*CEAP*, Clinical, Etiologic, Anatomic, and Pathophysiologic classification; *QoL*, quality of life.

Categorical data are shown as number (%).

Statements-reaching consensus are highlighted in bold in the tables.

**Table II:** Workshop 2 (W2): Medical and conservative management of chronic venous disease (*CVD*)—consensus statements, voting results, and key discussion points

Predefined questions	Statements	Vote % (n/N)		Key discussion points
Question 1 – W2Q1: What pharmacological treatments (eg, venoactive drugs) do you prescribe most frequently for CVD, and why?	**Once-daily diosmin-based venoactive drugs are widely used to relieve symptoms in patients with CVD (C0 to C6).**	**✓**	**94** (30/32)	- Pharmacological management represents a gold standard of conservative care in patients with CVD, particularly in the early stages or when interventional procedures are not indicated.
	**Once-daily diosmin is preferred for improving patient adherence. Dosage should exceed 450 mg and may reach up to 1000 mg. Duration of treatment should be at least 3 months.**	**✓**	**84** (26/31)	- Practice patterns regarding the type, dosing, and duration of treatment vary widely between countries and practitioners.
				- Key challenge: long-term adherence, patient education and tailoring treatment regimens to both symptom burden and patient expectations
Question 2 - W2Q2**:** What are the key challenges patients face in adhering to long-term conservative management?	**Adherence to long-term conservative treatment, especially compression, is limited by discomfort, comorbidities, cost, lifestyle factors, duration of the treatment and patient awareness.**	**✓**	**93** (29/31)	- Barriers to adherence: compression discomfort (particularly during warm seasons), cognitive/functional impairments in elderly, lack of understanding of the treatment’s rationale and high costs
				- Suggestions for long-term adherence: structured patient education and simpler regimens (eg, once-daily treatments or shorter initial cycles).
Question 3 – W2Q3: How do you evaluate the effectiveness of conservative treatments (like compression therapy) in your daily practice?	**Effectiveness of conservative treatment is primarily assessed through symptom relief and functional improvement of the patient using QoL assessment tools (such as CIVIQ 20 questionnaire).**	**✓**	**81** (25/31)	- Balance between patient-reported outcomes (PROMs) and objective measurements was discussed. - Agreement on the importance of combining subjective and objective methods to evaluate treatment response. - Symptom relief assessment (eg, CIVIQ–20) and routine use of objective evaluations (such as duplex ultrasound, leg circumference measurements, and ulcer size tracking) to ensure comprehensive monitoring.
	**Objective measurements such as ultrasound, measurement of ulcer size, diameter of the legs should be routinely used to evaluate the effectiveness of conservative treatment.**	**✓**	**90** (28/31)	
Question 4 – W2Q4: How do you combine pharmacological and nonpharmacological approaches for optimal symptoms control?	**For patients with CVD (C1-C6) any type of treatment modality should include venoactive drugs for optimal symptom relief.**	**✓**	**90** (28/31)	- Multifaceted approach needed:
				- Compression and lifestyle modification form the foundation of conservative care
				- Adjunct use of venoactive drugs (particularly diosmin-based agents) broadly supported for their additive effect on symptom relief, especially heaviness, pain, and cramps.
				- Combining strategies can increase patient adherence by reducing early symptom burden, particularly in C1 to C4 patients.

*CIVIQ-20*, Chronic Venous Insufficiency Questionnaire; *QoL*, quality of life.

Categorical data are shown as number (%).

Statements-reaching consensus are highlighted in bold in the tables.

**Table III:** Workshop 3 (W3): Surgical and interventional management of varicose vein (*VV*)—consensus statements, voting results, and key discussion points

Predefined questions	Statements	Vote % (n/N)		Key discussion points
Question 1 – W3Q1: What are the current indications for open surgery over minimally invasive surgery in VV management in your practice?	**Indications for vein surgery in VV management depend not only on medical issues but are highly influenced by the government medical policy, not being the same in the Western and Eastern countries.** **The patient's preference should be considered by the vascular professionals in each case.**	**✓** **✓**	**81** (26/32) **100** (32/32)	- Decision to perform open surgery vs a minimally invasive procedure rarely based on clinical factors alone. - Government health care policies, resource availability, reimbursement frameworks and geographical disparities significantly impact surgical approach. - Western Europe prefers minimally invasive; Eastern Europe/Central Asia often use open procedures due to limited access. - Patient preference as well as risks, recovery time, and outcomes are critical for shared decision-making in surgical planning.
Question 2 – W3Q2: How do you assess surgical risk in elderly patients with VV?	**The surgical risk in elderly patients with VVis not only based on the disease itself but depends on comorbidities that might influence the final outcome.**	**✓**	**97** (31/32)	- No universal definition for "Elderly"; variation by region and health context. - Age should not automatically preclude surgical intervention; comorbidities (such as cardiovascular disease, diabetes, and impaired mobility) are more relevant predictors of perioperative risk. - Strong support for individualized risk assessment and shared decision-making.
Question 3 – W3Q3: Are endovenous laser ablation (EVLA) and radiofrequency ablation (RFA) considered equivalent for first-line ablation of great saphenous vein reflux in terms of recurrence and patient-reported outcomes?	**EVLA and RFA are considered equivalent but the recurrence after any procedure in vein surgery does not only depend on the type of venous disease or the technical equipment, but even more on experience, technical skills, and indications for the procedure.**	**✓**	**93** (29/31)	- Both EVLA and RFA broadly accepted for first-line ablation, but regional preferences, availability of equipment, and training levels often dictate choice. - EVLA more widely used in several countries, reflecting recent trends. - Success and recurrence rates depend less on the technique itself than on operator experience, adherence to indications, and procedural consistency.
Question 4 – W3Q4: What criteria define surgical success in patients with VV?	**Surgical success in VV treatment is multifactorial, shaped by preoperative expectations, clearly defined procedural goals, postoperative patient compliance, and the rates of recurrence or need for reintervention.**	**✓**	**97** (29/30)	- Success cannot be assessed solely on technical endpoints (eg, vein occlusion or absence of reflux). - Patient expectations and satisfaction are critical markers (particularly symptom relief and QoL). - Goals should be clearly defined before intervention and revisited postoperatively. - Compliance with follow-up care and long-term monitoring for recurrence are essential. - Recurrence or need for reintervention should be interpreted within the full context of initial goals and clinical evolution.
Question 5 – W3Q5: How do you approach VV treatment before major surgery procedures?	**Taking into account available risk scores and venous thromboembolism (VTE) prophylaxis (eg, LMWH), conservative therapy (compression stockings + diosmin) should be the initial treatment of choice. In high-risk patients for superficial or deep vein thrombosis, VV intervention should be reconsidered, and when indicated, the least invasive technique possible should be selected.**	**✓**	**97** (31/32)	- Conservative treatment (ie, compression, venoactive drugs – including diosmin and LMWH) should be the default approach before major surgery. - Risk stratification (Caprini score) evaluates the risk of superficial or deep vein thrombosis. - Interventional treatment of varicose veins may be considered for high thrombotic risk but only using minimally invasive techniques (eg, endovenous thermal ablation) to limit trauma and promote rapid recovery. - Intervention should be timely planned and integrated within the broader perioperative strategy, in coordination with the surgical team managing the primary condition.

*LMWH*, Low-molecular-weight heparin; *QoL*, quality of life.

Categorical data are shown as number (%).

Statements-reaching consensus are highlighted in bold in the tables.

**Table IV:** Workshop 4 (W4): Medical and conservative management of varicose vein (*VV*)—consensus statements, voting results, and key discussion points

Predefined questions	Statements	Vote % (n/N)		Key discussion points
Question 1 – W4Q1: What pharmacological treatments are most effective in your practice for symptom relief?	**Only VADs can be prescribed from C0 to C6 grades, because they are safe and effective. However, for each pharmacological treatment, different criteria should be taken into account, such as inflammation, pain, mixed ulcers (C6), or rheumatological etiology of the disease**.	**✓**	**88** (29/33)	- VADs remain the most reliable pharmacological option for symptom relief across the CVD spectrum (C0-C6). - VADs described as both safe and effective, particularly when combined with compression therapy and lifestyle modifications. - While venoactive agents can be initiated broadly, individualized treatment decisions are essential. - Conservative treatment should be multimodal: compression therapy remaining the backbone, with drug treatment seen as a complementary strategy to reduce symptoms, delay progression, and improve quality of life.
Question 2 – W4Q2: How do you integrate VADs into the care pathway?	**According to guidelines and local protocols, and on an individual basis, VADs are used as part of the initial management of chronic venous disorders from C0 to C6 according to CEAP classification. They can both be used as monotherapy and in addition to conservative and interventional treatments.** **In phlebitis, thrombophlebitis, VADs should be prescribed.** In pregnant women, VADs should be prescribed from 6 months onward. **In patients with pelvic congestion syndrome, VADs should be prescribed as soon as the diagnosis is established.** VADs may also be prescribed in other venous-related conditions, such as hemorrhoids, migraine, postoperative edema, and post-traumatic edema.	**✓** **✓** X **✓** X	**77** (24/31) **97** (30/31) 58 (18/31) **97** (29/30) 74 (23/31)	- VADs should be part of CVD management across all CEAP stages (C0-C6); their utility should be individualized based on the severity of symptoms, presence of inflammation, and treatment setting. - These drugs can be used both as first-line monotherapy in early stages or in combination with compression or procedural treatments in more advanced disease. - Key areas of integration included initial management, as add-on to procedures, in phlebitis/thrombophlebitis, pregnancy, pelvic congestion syndrome and other indications (such as hemorrhoids, migraine with vascular features, postoperative, or post-traumatic edema). - Local protocols and reimbursement policies are key factors influencing practice variability across countries
Question 3 – W4Q3: Should VADs be continued after successful ablation to control residual symptoms (eg, heaviness and cramps)?	**VADs should be continued after successful ablation in patients with residual symptoms. Treatment duration should be for a minimum of 2 months following the procedure.**	**✓**	**100** (30/30)	- VADs should be continued in patients presenting with residual symptoms following successful ablation. - VADs are safe, well-tolerated, and beneficial in modulating postprocedural inflammation and improving microcirculatory function. - Vascular protective properties of VADs may support long-term outcomes by improving venous tone and reducing the risk of symptom recurrence. - Minimum duration for continued treatment for 2 months with longer durations possible depending on clinical response and disease severity. - Decision should be individualized based on symptom persistence and overall patient status.
Question 4 – W4Q4: Can diosmin-based venoactive therapy be considered as an alternative to procedural intervention in patients with low-symptom VV (eg, CEAP C2)?	**When contraindications to surgery or interventional procedures exist, or when the patient does not want to undergo surgery, diosmin-based venoactive therapy can be prescribed as an alternative. In this setting, it can be combined with compression therapy.**	**✓**	**87** (29/30)	- Diosmin-based therapy recognized as a pragmatic and individualized alternative in low-symptom C2 patients not candidates for intervention. - Procedural treatments (eg, ablation, phlebectomy) remain standard for treating saphenous reflux in symptomatic C2 patients. - Noninvasive strategies can be a valid alternative in selected cases when patients refuse intervention (ie, personal, cultural, or economic reasons) or in case of contraindications to surgery or interventional procedures - In these contexts, diosmin-based VADs were considered safe, well-tolerated, and potentially effective in reducing symptoms such as heaviness, swelling, or cramps. - Compression therapy should ideally be combined with VADs to optimize symptom control and slow progression of venous disease. - This approach should not be seen as curative nor equivalent to addressing underlying reflux, but as a symptom-relief strategy for those unable or unwilling to undergo definitive procedures.
Question 5 – W4Q5: Have you observed any efficacy variations between the dosage regimens of diosmin-based drugs?	**The optimal dose of pure diosmin is 600 mg. Dose-dependent effectiveness seems to be promising in advanced stages of CEAP classification (also by extrapolation of higher dosages used in hemorrhoidal condition management) but remains to be proven in chronic venous conditions.**	**✓**	**90** (28/31)	- Standard daily dose of pure diosmin (600 mg) is generally effective and well-tolerated across CVD stages. - Clinical rationale exists for exploring higher dosages, especially in advanced-stage patients with inflammation, mixed ulcers, or significant symptom burden. - In related venous conditions (such as hemorrhoids), higher dosages of diosmin or combination therapies are already in use and may inform extrapolations to CVD. - Robust comparative data in CVD are missing; additional clinical studies needed to further assess dose-response relationships, particularly in long-term use and for specific CEAP stages. - Standard dosing should be used while considering individualized adjustments in select cases under close clinical supervision.
Question 6 – W4Q6: What are the most common barriers to conservative treatment adherence?	**The main barriers to treatment adherence are the lack of strong scientific evidence (eg, RCTs), insufficient physician awareness of the benefits of treatment, limited reimbursement, and inadequate patient self-education in CVD**.	**✓**	**93** (28/30)	- Barriers affecting adherence to conservative therapies include: lower confidence among physicians and policymakers for some conservative measures due to limited evidence. Variable physician awareness and education, with some clinicians underestimating the value of noninvasive treatments. Reimbursement policies (or lack of) as a substantial obstacle in several countries, particularly for compression garments and VADs. Patient self-education and limited understanding of CVD as a chronic, evolving disease – this may lead to early discontinuation of treatment when symptoms improve. Cultural beliefs, seasonal discomfort with compression wear, and limited follow-up mechanisms as contributing factors in some regions. - The importance of patient empowerment, ongoing physician education, and policy-level advocacy to address these structural and behavioral gaps.

*CEAP*, Clinical, Etiologic, Anatomic, and Pathophysiologic classification; *CVD*, chronic venous disease; *RCTs*, randomized controlled trials; *VADs*, venoactive drugs.

Categorical data are shown as number (%).

Statements-reaching consensus are highlighted in bold in the tables.

The second step of the process was the expert panel composition. Thirty-eight experts in phlebology were selected to participate in the consensus process. The expert panel was selected according to predefined professional and scientific criteria. Experts were required to have substantial clinical experience in venous diseases, be actively practicing and board-certified, and demonstrate recognized scientific expertise through research achievements, publications, scientific presentations, or leadership in the scientific societies. To strengthen the validity and representativeness of the consensus, the panel included experts from different countries and geographic regions.

The third step aimed at organizing the workshop discussions. Each expert was assigned to one of the four thematic working groups before the meeting based on their respective specialty, in order to ensure balanced group in terms of clinical expertise diversity and group size (approximately 10 participants/group). The predefined questions (see Supplementary Table, online only) were shared with the participant in advance. Each group participated in a structured 3-hour workshop session, moderated by an experienced clinician (academic professor, department head in phlebology, or vascular surgery). During these workshops, each question was discussed in detail, allowing participants to share perspectives and insights on the basis of their clinical experience and national practice patterns. Following the discussion, each group collaboratively drafted one or more key statements per question, summarizing their collective position or proposed recommendation.

The fourth and final step consisted of plenary voting to assess the degree of agreement. Statements were then compiled and presented to the entire panel of 38 experts for electronic voting during a plenary session, using the Slido platform. Voting was anonymous and conducted in real time. Participants were asked to rate each statement using a 5-point Likert scale ranging from 1 (strongly disagree) to 5 (strongly agree).

A consensus was defined a priori as agreement by ≥75% of respondents rating a statement as 4 (agree) or 5 (strongly agree).

After the vote, all statements were reviewed by the faculty to enhance clarity and improve English language expression, without altering their original meaning.

## Results

A total of 20 predefined clinical questions were discussed across the four thematic workshops, leading to the formulation of 34 draft consensus statements, which were subsequently submitted to a vote in plenary session.

Out of the 34 statements, 30 (88%) reached the predefined consensus threshold (≥75% agreement), whereas four statements did not reach consensus.

Results are presented through Workshop in Table I, Table II, Table III, Table IV. For each predefined question, the tables report the statement drafted by each workshop group and submitted to the full expert panel for voting during the plenary session, together with the level of agreement, and the main points raised during discussion. Statements-reaching consensus are highlighted in bold in the tables, whereas statements that did not are also reported with their corresponding agreement levels. Some questions resulted in more than one statement, addressing different discussion points within the same clinical topic.

Overall, workshop 1 generated 12 draft statements, of which 10 reached consensus. Workshops 2 and 3 each generated 6 draft statements, all of which reached consensus. Workshop 4 generated 10 draft statements, of which 8 reached the consensus threshold during the plenary vote.

## Discussion

### Principal findings

The management of venous disease includes a broad range of therapeutic options that are often adapted to local practices, patient characteristics, symptom burden, and available resources. Despite extensive literature, variability in clinical practice remains substantial, supporting the value of expert consensus to guide decision-making, particularly in complex or controversial situations. This multinational Delphi-based consensus initiative aimed to identify areas of agreement and divergence among international phlebology specialists across four thematic workshops. Overall, 34 statements were formulated, of which 30 (88%) reached consensus, providing a broad evaluation of current strategies for the management of CVD and VV across diverse health care settings. Key areas of agreement included the importance of symptoms and QoL in therapeutic decision-making, the preference for endovenous techniques when feasible, and the widespread but heterogeneous use of compression therapy and VADs, with persistent challenges regarding adherence and access. These findings are broadly consistent with the European Society for Vascular Surgery (ESVS) 2022 guidelines and the 2022/2023 Society for Vascular Surgery-American Venous Forum-American Vein and Lymphatic Society guidelines, particularly regarding the patient-centered decision-making, duplex-based assessment, and endovenous ablation for symptomatic truncal reflux when expertise and technology are available. However, the present consensus also reflects real-world differences between health care systems, including variability in access to procedures, the role and duration of compression therapy, and the availability, regulatory status, and perceived level of evidence for VADs. Thus, this consensus complements existing guidance by highlighting areas of shared practice while acknowledging the practical heterogeneity that continues to influence venous disease management internationally.

#### Workshop 1: Surgical and interventional management of CVD

The panel strongly endorsed the use of CEAP classification and symptom severity to guide interventions to treat CVD. Intervention was recommended primarily for symptomatic patients with CEAP classes C4 to C6 disease and in selected highly symptomatic C3 cases, guided by imaging and clinical impact. This patient-centric, symptom-driven approach aligns with ESVS 2022 guideline, which places patient-reported outcomes at the core of decision-making and with earlier publications emphasizing the importance of individualized anatomical assessment.^12^^,^^18^^,^^19^ However, the role of open surgery has become limited dependent mostly on economic conditions, whereas the endovascular techniques are now preferred more often.^19^

Post-thrombotic syndrome (PTS) refers to chronic manifestations of venous insufficiency following an episode of deep vein thrombosis, being an important and frequent long-term adverse event of proximal deep vein thrombosis within 2 years in 20% to 50% of patients.^20^ The management of PTS remains challenging, particularly regarding the selection of interventional strategies. For symptomatic iliac vein lesions and post-thrombotic obstruction, endovascular procedures were supported as first-line therapy. Evidence shows clinical and QoL benefits, though patency is lower in post-thrombotic cases compared with nonthrombotic iliac vein lesions, which is an important consideration for patient counseling.^12^

Hybrid or open surgery may still be appropriate in certain cases, particularly for complex iliocaval obstructions.

Consensus was not reached on universal eligibility of elderly patients for intervention, underlining the importance of individualized risk assessment. Although cardiac risk scores such as EuroSCORE, a tool used to predict a patient's risk of death following heart surgery, have occasionally been extrapolated to venous procedures, they are not validated in this setting. Recent evidence indicates that patients with CVD carry a substantially increased burden of cardiovascular morbidity and mortality, yet routine cardiovascular risk assessment is rarely performed.^21^ Onida and Davies^21^ have advocated for incorporating standardized cardiovascular risk stratification (eg, QRISK3, a tool used in primary care to estimate a healthy adult's percentage risk of developing cardiovascular disease over the next 10 years) into the management of venous disease, as this could improve personalization of treatment and long-term outcomes.^21^ Conservative alternatives therefore remain critical for high-risk patients, and their inclusion in treatment pathways reflects real-world practice.

Finally, the definition of successful outcomes remains debated. The panel emphasized a multifactorial definition of procedural success, integrating predefined objectives, symptom relief, QoL, and need for reintervention. The role of CEAP classification changes and objective follow-up timing requires further validation.^22^

This aligns with the use of patient-reported outcome measures (PROMs) such as Venous Insufficiency Epidemiological and Economic Study-Quality of Life/Symptom and Chronic Venous Insufficiency Questionnaire-20, which are validated instruments in CVD.^18^ This is supported by clinical data: in a prospective study of patients undergoing conventional surgery for CVD, Matei et al^23^ reported significant functional and psychosocial QoL improvement, even in the early postoperative period.

#### Workshop 2: Medical and conservative management of CVD

Compression therapy remains a cornerstone for symptom control and ulcer healing.^12^^,^^24^ However, adherence remains problematic, hindered by discomfort, seasonal intolerance, cost, and lack of patient education.^25^ These barriers underscore the need for improved strategies to enhance patient compliance and ensure more effective treatment outcomes.

A Cochrane review concluded that phlebotonics probably reduce edema and improve some symptoms.^26^ In clinical practice, once-daily formulations are generally preferred because simplified regimens may help improve adherence.

Remaining uncertainties include optimal dosing and duration of VADs treatment, ranging from 2 to 6 months, timing of intervention in minimally symptomatic disease, and the role of pharmacological therapy in pregnancy, where conservative approaches remain preferable.

The panel also underlined the complementary role of pharmacological and nonpharmacological strategies in the management of CVD, reflecting current practice patterns in Europe. Evaluation methods for conservative treatment are evolving, with an increasing focus on combining subjective outcomes (eg, QoL questionnaires) and objective measures (eg, ultrasound parameters and leg circumference). This approach is consistent with recent consensus recommendations and highlights the need for standardized assessment frameworks in future studies.^12^^,^^27^

Finally, broad consensus was reached regarding the use of diosmin-based VADs, often combined with compression, for symptom relief, across CEAP classes. This reflects international guidance.^28^

#### Workshop 3: Surgical and interventional management of VV

This workshop highlighted variability in practice between open and minimally invasive treatments. The influence of local policy and health care system structures was considered critical, particularly in Eastern Europe and low-resource settings. Such disparities affect care equity and reinforce the importance of shared decision-making, as underlined in international guidelines.^12^

Surgical risk assessment, especially in elderly patients, was recognized as multifactorial, requiring evaluation of comorbidities rather than age alone.

Equivalence between endovenous laser ablation (EVLA) and radiofrequency ablation was acknowledged, consistent with multiple meta-analyses showing similar outcomes in terms of symptom relief and recurrence.^29^^,^^30^ A more recent systematic review confirmed that both techniques provide comparable occlusion rates with radiofrequency ablation demonstrating slightly higher long-term patency and fewer minor complications, whereas EVLA was associated with less pigmentation.^31^

In addition, other evidence suggests EVLA may provide durable long-term results, whereas nonthermal techniques such as cyanoacrylate or mechanochemical ablation avoid thermal injury and the need for tumescent anesthesia. Foam sclerotherapy remains a low-cost option, often used for retreatment.^32^

Definitions of success were recognized as multifactorial, incorporating symptom relief, recurrence rates, and patient expectations. Operator expertise and local availability of techniques were also highlighted as decisive factors.

The integration of patient preferences and structured risk assessment before major procedures, particularly for the prevention of venous thromboembolism, was highlighted as a consensus point. This approach is consistent with recent recommendations from the ESVS, which emphasize individualized decision-making and perioperative risk management.^12^

#### Workshop 4: Medical and conservative management of VV

The panel confirmed that VADs, particularly diosmin-based formulations, have a role across CEAP classes, and may be prescribed in various venous conditions.^33^ This broad use is reflected in international guidelines.^28^^,^^34^ Their potential as an alternative to intervention in low-symptom patients was pointed out, particularly when surgery is declined or contraindicated. Evidence remains limited, but real-world data suggest symptom benefit.^34^ A high level of agreement was also reached regarding the continuation of VADs after ablation, to manage residual symptoms. However, the optimal duration remains uncertain and may depend on CEAP stage. Regarding dosing, 600 mg once daily was considered standard, whereas higher doses (used in other indications such as hemorrhoidal disease) were noted as potentially beneficial in advanced venous stages, though further trials are needed to support this extrapolation.

Compression therapy was again endorsed as a core element of conservative management, though adherence challenges persist. Systematic reviews have identified barriers including discomfort, cost, and difficulties with application, underscoring the need for tailored interventions and physician engagement.^35^, ^36^, ^37^

### Strengths and limitations

Strengths include the multinational and multidisciplinary composition of the panel, allowing integration of diverse clinical perspectives. Panel was composed of experienced vascular and phlebology experts. The structured Delphi-like methodology, combining predefined questions, moderated group discussions, and anonymous voting, minimized the influence of dominant individuals and strengthened the robustness of the consensus. High consensus rates across most statements support the consistency of the findings.

Limitations include reliance on expert opinion where randomized controlled trials are lacking, incomplete global representation, heterogeneity in reimbursement and access, and absence of patient participation. Post-stenting anticoagulation was not evaluated, as thrombotic venous disease was beyond the primary scope of this consensus, which focused on chronic venous insufficiency and VV.

### Future research directions

Future research in venous disease should focus on improving the consistency, quality, and clinical relevance of available evidence. Although validated instruments such as Chronic Venous Insufficiency Questionnaire-20 and Venous Insufficiency Epidemiological and Economic Study-Quality of Life/Symptom provide robust and standardized outcome assessment, their routine implementation in high-volume clinical practice may be limited by practical constraints, highlighting the need for simpler PROMs. Further clinical trials are needed to determine the optimal dosing and duration of VADs, as current data remain heterogeneous and often limited to short-term assessments.

Strategies to enhance adherence to compression therapy should be addressed. In addition, comparative long-term studies evaluating outcomes of endovascular procedures, particularly between nonthrombotic iliac vein lesions and PTS, are essential to refine patient selection and confirm long-term safety of interventions. The development and validation of venous-specific risk stratification models could further enable a more individualized approach to management and a better prediction of therapeutic benefit across chronic venous disorders.

Beyond clinical trials, innovation will shape future practice. Artificial intelligence is emerging as a transformative tool in venous diagnostics, with potential to enhance accuracy, streamline workflows, and enable personalized management. Matei et al^38^ recently underlined its potential to address current challenges in venous diagnostics and improve patient outcomes.

## Conclusions

This multinational consensus provides pragmatic, patient-oriented recommendations for CVD and VV management that are applicable across diverse health care settings. It highlights a shift from anatomy-driven to symptom- and QoL-driven care. It also confirms the equivalence of thermal ablation techniques and emphasizes the complementary roles of VADs, such as diosmin, and compression. This work acknowledges ongoing challenges with adherence and patient selection. The consensus also highlighted regional disparities, both in practice and policy, which continue to influence treatment accessibility and modality choice. Future research should prioritize long-term outcomes, conservative management strategies, and integration of PROMs to harmonize venous care globally.

## Author Contributions

Conception and design: VC, NI

Analysis and interpretation: S-CM, ST, NI

Data collection: S-CM, NN, MP, VS, ST, VC, YM, JE, NI

Writing the article: S-CM, ST, YM, JE

Critical revision of the article: S-CM, NN, MP, VS, ST, VC, NI

Final approval of the article: S-CM, NN, MP, VS, ST, VC, YM, JE, NI

Statistical analysis: Not applicable

Obtained funding: Not applicable

Overall responsibility: S-CM

## Funding

The working group was supported by a research grant from Innotech International. The sponsor supported manuscript writing and data collection but was not involved in the study design, analysis, or interpretation of the data. The sponsor was not involved in the decision to submit the manuscript for publication.

## Ethics approval

No ethics committee approval was required, as this was a noninterventional, nonclinical consensus initiative and did not involve patient-level data collection. All workshop members approved the use of their contributions.

## Disclosures

During the conduct of this work, N.I. and V.C. received a consulting fees from Innotech International. M.P., N.N., S.M., and N.I. received research support from the sponsor for lecture speaking. Y.M. and J.E. are employees of Innotech International. V.S. and S.T. have no conflicts of interest.

## References

[1] Eberhardt R.T., Raffetto J.D. (2014). Chronic venous insufficiency. Circulation.

[2] Ortega M.A., Fraile-Martinez O., Garcia-Montero C. (2021). Understanding chronic venous disease: a critical overview of its pathophysiology and medical management. J Clin Med.

[3] Robertson L., Evans C., Fowkes F.G. (2008). Epidemiology of chronic venous disease. Phlebology.

[4] Salim S., Machin M., Patterson B.O., Onida S., Davies A.H. (2021). Global epidemiology of chronic venous disease: a systematic review with pooled prevalence analysis. Ann Surg.

[5] Zolotukhin I.A., Seliverstov E.I., Shevtsov Y.N. (2017). Prevalence and risk factors for chronic venous disease in the general Russian population. Eur J Vasc Endovasc Surg.

[6] Vuylsteke M.E., Thomis S., Guillaume G. (2015). Epidemiological study on chronic venous disease in Belgium and Luxembourg: prevalence, risk factors, and symptomatology. Eur J Vasc Endovasc Surg.

[7] Santiago F. (2023). Quality of life in chronic venous disease: bridging the gap between patients and physicians. Clin Drug Investig.

[8] Nicolaides A.N., Labropoulos N. (2019). Burden and suffering in chronic venous disease. Adv Ther.

[9] Kienzl P., Deinsberger J., Weber B. (2024). Chronic venous disease: pathophysiological aspects, risk factors, and diagnosis. Hamostaseologie.

[10] Vuylsteke M.E., Colman R., Thomis S. (2018). An epidemiological survey of venous disease among general practitioner attendees in different geographical regions on the globe: the final results of the vein consult Program. Angiology.

[11] Grant Y., Onida S., Davies A. (2017). Genetics in chronic venous disease. Phlebology.

[12] De Maeseneer M.G., Kakkos S.K., Aherne T. (2022). Editor's choice–European Society for Vascular Surgery (ESVS) 2022 clinical practice guidelines on the management of chronic venous disease of the lower limbs. Eur J Vasc Endovasc Surg.

[13] Gloviczki P., Comerota A.J., Dalsing M.C. (2011). The care of patients with varicose veins and associated chronic venous diseases: clinical practice guidelines of the Society for Vascular Surgery and the American Venous Forum. J Vasc Surg.

[14] Carman T.L., Al-Omari A. (2019). Evaluation and management of chronic venous disease using the foundation of CEAP. Curr Cardiol Rep.

[15] Santler B., Goerge T. (2017). Chronic venous insufficiency - a review of pathophysiology, diagnosis, and treatment. J Dtsch Dermatol Ges.

[16] Davies A.H. (2019). The seriousness of chronic venous disease: a review of real-world evidence. Adv Ther.

[17] Ruquaya S. (2025). Predictors of recurrent varicose veins: a systematic review of risk factors contributing to recurrence after treatment. Int J Med Sci Clin Res Rev.

[18] Lamping D.L., Schroter S., Kurz X., Kahn S.R., Abenhaim L. (2003). Evaluation of outcomes in chronic venous disorders of the leg: development of a scientifically rigorous, patient-reported measure of symptoms and quality of life. J Vasc Surg.

[19] Raju S., Neglen P. (2009). Percutaneous recanalization of total occlusions of the iliac vein. J Vasc Surg.

[20] Visona A., Quere I., Mazzolai L. (2021). Post-thrombotic syndrome. Vasa.

[21] Onida S., Davies A.H. (2025). Cardiovascular risk assessment in venous disease?. Lancet.

[22] Gloviczki P., Lawrence P.F., Wasan S.M. (2023). The 2022 Society for Vascular Surgery, American Venous Forum, and American Vein and Lymphatic Society clinical practice guidelines for the management of varicose veins of the lower extremities. Part I. Duplex scanning and treatment of superficial truncal reflux: endorsed by the Society for Vascular Medicine and the International Union of Phlebology. J Vasc Surg Venous Lymphat Disord.

[23] Matei S.C., Radu-Teodorescu D., Murariu M.S., Dumitru C.Ș., Olariu S. (2024). Cryostripping versus conventional safenectomy in chronic venous disease treatment: a single Center retrospective cohort Study. Chirurgia (Bucur).

[24] Stanek A., Mosti G., Nematillaevich T.S. (2023). No more venous ulcers-what more can we do?. J Clin Med.

[25] Rabe E., Partsch H., Hafner J. (2018). Indications for medical compression stockings in venous and lymphatic disorders: an evidence-based consensus statement. Phlebology.

[26] Martinez-Zapata M.J., Vernooij R.W., Simancas-Racines D. (2020). Phlebotonics for venous insufficiency. Cochrane Database Syst Rev.

[27] Aloi T.L., Camporese G., Izzo M., Kontothanassis D., Santoliquido A. (2019). Refining diagnosis and management of chronic venous disease: outcomes of a modified Delphi consensus process. Eur J Intern Med.

[28] Nicolaides A., Kakkos S., Baekgaard N. (2018). Management of chronic venous disorders of the lower limbs. Guidelines according to scientific evidence. Part I. Int Angiol.

[29] He G., Zheng C., Yu M.A., Zhang H. (2017). Comparison of ultrasound-guided endovenous laser ablation and radiofrequency for the varicose veins treatment: an updated meta-analysis. Int J Surg.

[30] Lalam N., Chandrashekhara P., Bhagavan K. (2025). A comparative study of endovenous laser ablation and radiofrequency ablation in the management of varicose veins. Int Surg J.

[31] Jiang W., Liang Y., Long Z. (2024). Endovenous radiofrequency ablation vs laser ablation in patients with lower extremity varicose veins: a meta-analysis. J Vasc Surg Venous Lymphat Disord.

[32] Shaprynskyi V.O., Shaprynskyi V.V., Semenenko N.V. (2023). Management of miniinvasive treatment of primary varicose superficial veins. Wiad Lek.

[33] Gloviczki M.L., Kakkos S.K., Urbanek T., Chuback J., Nicolaides A. (2025). The role of venoactive compounds in the treatment of chronic venous disease. J Vasc Surg Venous Lymphat Disord.

[34] Gloviczki P., Lawrence P.F., Wasan S.M. (2024). The 2023 Society for Vascular Surgery, American Venous Forum, and American Vein and Lymphatic Society clinical practice guidelines for the management of varicose veins of the lower extremities. Part II: endorsed by the Society of Interventional Radiology and the Society for Vascular Medicine. J Vasc Surg Venous Lymphat Disord.

[35] Attaran R.R., Edwards M.L., Arena F.J. (2025). 2025 SCAI clinical PrACTICE GUidelines for the management of chronic venous disease: this statement was endorsed by the Society for Vascular Medicine (SVM). J Soc Cardiovasc Angiogr Interv.

[36] Bar L., Brandis S., Marks D. (2021). Improving adherence to wearing compression stockings for chronic venous insufficiency and venous leg ulcers: a scoping review. Patient Prefer Adherence.

[37] Tan M., Urbanek T., Rabe E. (2024). Compression therapy in the management of varicose veins. Phlebology.

[38] Matei S.C., Olariu S., Ungureanu A.M., Malita D., Petrascu F.M. (2025). Does artificial intelligence bring new insights in diagnosing phlebological diseases?-A systematic review. Biomedicines.

