# Supplementary material for: Optimizing the management of venous disease: Expert consensus from a multinational panel of phlebology specialists
Source: J Vasc Surg Venous Lymphat Disord. 2026 Aug 4;14(6):102593. doi: 10.1016/j.jvsv.2026.102593 (PMC13598173; doi:10.1016/j.jvsv.2026.102593)
Supplement: Supplementary file 1 [file mmc1.docx]

# *Supplemental Table I*: List of predefined questions

**Group 1 - CVD – Surgical & Interventional strategies**

1. How do you decide among hybrid, open surgery, and endovascular techniques in post-thrombotic syndrome (PTS)?
2. Are there patient populations for whom intervention should be avoided?
3. What is the alternative for those patients?
4. Which anatomical or clinical criteria most influence your treatment choice? (CEAP classification, duplex findings, symptom severity, etc.)?
5. How do you define a successful outcome after surgery or intervention in PTS patients?

**Group 2 - CVD – Medical & Conservative management**

1. What pharmacological treatments (e.g., venoactive drugs) do you prescribe most frequently for CVD, and why?
2. What are the key challenges patients face in adhering to long-term conservative management?
3. How do you evaluate the effectiveness of conservative treatments (like compression therapy) in your daily practice?
4. How do you combine pharmacological and non-pharmacological approaches for optimal symptoms control?

**Group 3 - Varicose veins – Surgical & Interventional strategies**

1. What are the current indications for open surgery over minimally invasive surgery in varicose vein management in your practice?
2. How do you assess surgical risk in elderly patients with varicose veins?
3. Are EVLA and RFA considered equivalent for first-line ablation of great saphenous vein reflux in terms of recurrence and patient-reported outcomes?
4. What criteria define surgical success in varicose vein patients?
5. How do you approach varicose vein treatment before major surgery procedures?

**Group 4 - Varicose veins – Medical & conservative management**

1. What pharmacological treatments are most effective in your practice for symptom relief?
2. How do you integrate venoactive drugs into the care pathway?
3. Should venoactive drugs be continued after successful ablation to control residual symptoms (e.g., heaviness, cramps)?
4. Can diosmin-based venoactive therapy be considered as an alternative to procedural intervention in low-symptom varicose vein patients (e.g., CEAP C2)?
5. Have you observed any efficacy variations between the dosage regimens of diosmin-based drugs?
6. What are the most common barriers to conservative treatment adherence?
